# Clinically Relevant Pharmacogenomic Variant Frequencies in Kazakh, Russian, and Uzbek Population Groups Residing in Kazakhstan

**DOI:** 10.3390/biology15171477

**Published:** 2026-09-01

**Authors:** Zhassulan Zhaniyazov, Akmaral Kulatayeva, Aikorkem Mustafayeva, Assel Aulbekova, Nazym Altynova, Gulnur Zhunussova, Madina Abdullayeva, Salimat Ryspaeva, Rauash Mangazbayeva, Beimbet Daribayev, Saltanbek Mukhambetzhanov, Leyla Djansugurova

**Affiliations:** 1 RSE “Institute of Genetics and Physiology” of the Science Committee of the Ministry of Science and Higher Education of the Republic of Kazakhstan, 93 Al-Farabi Avenue, Almaty 050060, Kazakhstan; mustafaeva.aikorkem08@gmail.com (A.M.); madessin7@gmail.com (M.A.);; 2 Faculty of Biology and Biotechnology, Al-Farabi Kazakh National University, 71 Al-Farabi Avenue, Almaty 050040, Kazakhstan; 3 Faculty of Information Technology and Artificial Intelligence, Al-Farabi Kazakh National University, 71 Al-Farabi Avenue, Almaty 050040, Kazakhstan; 4 Department of Chemical and Biochemical Engineering, Geology and Oil-Gas Business Institute Named After K. Turyssov, Satbayev Kazakh National Research Technical University (Satbayev University), 22 Satbayev Street, Almaty 050013, Kazakhstan; s.ryspayeva@satbayev.university (S.R.);; 5 Department of Physics and Mathematics, Shakarim University, 20A Glinka Street, Semey 071410, Kazakhstan

**Keywords:** pharmacogenomics, pharmacogenetic variants, allele frequency, genotype frequency, ClinPGx, gnomAD, Kazakhstan, Central Asia, precision medicine

## Abstract

Central Asian populations remain underrepresented in pharmacogenomic research, limiting the availability of population-specific data for genotype-informed prescribing and precision medicine. This study analyzed clinically relevant pharmacogenomic variant frequencies in Kazakh, Russian, and Uzbek population groups residing in Kazakhstan using genome-wide genotype data from 1301 individuals: Kazakh (*n* = 1111), Russian (*n* = 156), and Uzbek (*n* = 34). ClinPGx, a PharmGKB-based clinical annotation framework that prioritizes variant–drug associations according to levels of evidence, was used to select variants with evidence levels 1A, 1B, and 2A. In total, 112 directly genotyped variants were retained for population-specific allele and genotype frequency analysis. All 112 variants were queried against the gnomAD v4.1 genome and exome reference datasets. Of these, matching allele-frequency data for the predefined reported allele were available in at least one of the two gnomAD datasets for 103 variants, whereas for 9 variants the VEP-based query did not return a matching gnomAD frequency for that allele. Frequencies were reported for the same predefined reported allele across all groups, and differences between the study groups were assessed using 95% confidence intervals, Fisher’s exact tests, and false discovery rate correction. Genotype counts and the proportions of individuals carrying at least one copy of the reported allele were also summarized for all selected variants. Several pharmacogenomic variants showed population-specific frequency patterns, including *NUDT15* rs116855232, *SLCO1B1* rs4149056, *VKORC1* rs9934438, and *UGT1A1* rs10929302. Comparison with gnomAD showed that the observed frequencies were variant-specific and could not be consistently approximated by a single broad genetic ancestry group. Reference-based population structure analysis provided additional ancestry context and supported separate reporting by population group. The study did not evaluate clinical outcomes or make individual prescribing recommendations, and the small Uzbek sample size limits the precision of frequency estimates for this group, particularly for rare variants. Overall, this study provides a clinically prioritized pharmacogenomic frequency resource for underrepresented population groups in Kazakhstan and supports broader Central Asian representation in pharmacogenomic implementation research.

## Simple Summary

Central Asian populations remain poorly represented in pharmacogenomic research, limiting the availability of population-specific information for precision medicine. In this study, we analyzed 112 directly genotyped, clinically relevant pharmacogenomic variants in 1301 Kazakh, Russian, and Uzbek individuals residing in Kazakhstan. The frequencies of these variants were compared among the three population groups and with the contemporary gnomAD v4.1 genome and exome reference datasets. Of the 112 variants, matching reference allele-frequency data for the predefined reported allele were available in at least one of the two gnomAD datasets for 103 variants; no corresponding gnomAD frequency was available for the remaining 9 variants. Several variants showed notable population-specific patterns, including variants in *NUDT15*, *SLCO1B1*, *VKORC1*, and *UGT1A1*, which are relevant to thiopurine toxicity, statin-associated adverse effects, warfarin dose variability, and irinotecan-related toxicity. The comparisons showed that the observed frequency patterns could not be consistently represented by a single broad global reference group. These findings provide an updated pharmacogenomic frequency resource for Kazakhstan and highlight the need for larger, more balanced, and clinically integrated studies in Central Asia.

## 1. Introduction

Pharmacogenomics is an important component of precision medicine because inherited genetic variation can influence drug metabolism, transport, therapeutic efficacy, toxicity, and dosing requirements. Clinically relevant pharmacogenomic variants are found in genes encoding drug-metabolizing enzymes, membrane transporters, and drug targets. With the expansion of pharmacogenomic evidence, curated resources such as PharmGKB and ClinPGx have become important for organizing variant–drug associations, assigning levels of clinical evidence, and prioritizing markers with potential clinical relevance [1,2]. Because many pharmacogenomic recommendations are gene–drug specific, population-level data on clinically relevant variant frequencies are needed to estimate the potential impact of pharmacogenomic testing across diverse healthcare settings.

The clinical implementation of pharmacogenomics is increasingly supported by evidence-based prescribing guidelines, including recommendations developed by the Clinical Pharmacogenetics Implementation Consortium (CPIC) and the Dutch Pharmacogenetics Working Group (DPWG). These guidelines translate selected gene–drug associations into recommendations relevant to drug dosing, toxicity prevention, and therapeutic response. However, the population-level relevance of such recommendations depends partly on the local frequency of clinically important pharmacogenomic variants. For countries and regions with limited pharmacogenomic data, including Kazakhstan and Central Asia, population-specific frequency resources are therefore needed before broader implementation strategies can be designed.

A major consideration in pharmacogenomics is that the frequencies of clinically relevant alleles differ substantially across populations. These differences can influence the expected prevalence of pharmacogenomic phenotypes and the potential public-health impact of genotype-guided prescribing. Large reference projects, including the 1000 Genomes Project, have demonstrated extensive genetic diversity across global populations, while large-scale pharmacogenomic studies in biobanks have further shown that pharmacogenomic allele and phenotype frequencies vary between biogeographic groups [3,4,5]. Therefore, population-specific pharmacogenomic frequency data are needed, especially for populations that remain underrepresented in global genomic and pharmacogenomic resources. This issue is particularly relevant for Central Asian populations, which are often absent from large pharmacogenomic reference datasets or represented only indirectly through broad continental population categories [6,7].

Kazakhstan provides an important setting for population-informed pharmacogenomic research because it is a multiethnic country located between Europe and Asia. According to the 2021 National Population Census, Kazakhs constitute the largest ethnic group in the country, while Russians and Uzbeks represent the second and third largest groups, accounting for 70.4%, 15.5%, and 3.2% of the population, respectively [8]. In this context, reporting a single combined national estimate may mask population-specific pharmacogenomic patterns. Analysis of major population groups residing in Kazakhstan within the same genotyping and analytical framework can provide a more informative description of local pharmacogenomic diversity and may help support future precision medicine initiatives in the country.

At the same time, population labels in pharmacogenomic studies require careful interpretation. Self-reported population identity reflects social, cultural, and genealogical information, but it does not fully capture individual genetic ancestry or admixture. This consideration is especially relevant in Central Asia, where historical population movements and admixture have contributed to complex patterns of genetic variation. In the present study, self-reported Kazakh, Russian, and Uzbek group labels were used for population-level frequency reporting, while reference-based PCA was added to provide ancestry context rather than to redefine individual identity.

Previous studies have begun to describe genetic and pharmacogenetic variation in Kazakh populations. Iskakova and colleagues analyzed ADME-related SNPs in Kazakhs from Kazakhstan and compared their distribution with global populations [9]. More recent studies have examined pharmacogenetic predictors of drug response in clinical cohorts from Kazakhstan, including metformin response in Kazakh patients with metabolic syndrome and type 2 diabetes [10]. In parallel, population-genomic resources for Kazakhs have expanded, including recent large-scale genotype data from healthy Kazakh individuals [11]. However, pharmacogenomic data from Kazakhstan remain limited, and less information is available on clinically prioritized pharmacogenomic variants across multiple population groups residing in Kazakhstan using the same analytical framework. Contemporary large-scale resources such as gnomAD provide allele-frequency data across broad genetic ancestry groups and offer a useful reference context for populations that remain underrepresented in global genomic datasets [12,13].

The present study aimed to characterize and compare clinically relevant pharmacogenomic variant frequencies in Kazakh, Russian, and Uzbek population groups residing in Kazakhstan. We hypothesized that the frequencies of clinically relevant variants would differ among the three groups and that their patterns would not be adequately represented by a single broad global reference population. This study was intended to provide a population-specific pharmacogenomic frequency resource for Kazakhstan and to improve the representation of Central Asian populations in pharmacogenomic research.

## 2. Materials and Methods

### 2.1. Study Cohort and Sample Source

The study included genome-wide genotype data from 1301 individuals representing three self-reported population groups residing in Kazakhstan: Kazakh (*n* = 1111), Russian (*n* = 156), and Uzbek (*n* = 34). Population group assignment was based on self-reported population identity recorded in the available sample metadata. DNA samples were obtained from the biobank of the Institute of Genetics and Physiology. All analyses were performed separately for each population group.

The Local Ethics Committee of the Institute of Genetics and Physiology, Committee of Science, Ministry of Science and Higher Education of the Republic of Kazakhstan approved the study (Protocol No. 3 dated 30 October 2023). All participants provided written informed consent for the use of their biological samples and genetic data for research purposes.

### 2.2. Genotyping and Quality Control

Genotyping was performed using the Illumina Global Screening Array v3 on the Illumina iScan platform (Illumina, Inc., San Diego, CA, USA). Genotype calling and initial data processing were performed using Illumina GenomeStudio 2.0 (Illumina, Inc., San Diego, CA, USA). Genotype data were exported in PLINK binary format, and variant positions corresponded to the GRCh38/hg38 genome build.

Sample- and variant-level quality control was performed using PLINK v1.9 [14]. The analysis was restricted to directly genotyped variants; genotype imputation was not performed. Sample-level quality control included assessment of genotype call rate and relatedness. Relatedness between individuals was assessed using PLINK identity-by-descent analysis, with a PI_HAT threshold of 0.25 used to flag potentially related pairs for review. Final sample inclusion was determined after application of the genotype call-rate and relatedness criteria.

For genome-wide population-structure analysis, autosomal variants on chromosomes 1–22 were used, and missingness filters were applied using --mind 0.02 and --geno 0.02. Additional variant filtering and linkage-disequilibrium pruning were applied before PCA as described below. Because all samples were processed using the same genotyping platform and analytical workflow, no separate batch-specific correction was applied.

Clinically annotated pharmacogenomic variants were intersected with the available genotype dataset by rsID. No minor allele-frequency threshold was applied to the pharmacogenomic variant set because clinically relevant variants may be rare.

### 2.3. Population Structure Analysis

Principal component analysis (PCA) was performed to provide ancestry context for the pharmacogenomic frequency analysis. Genome-wide autosomal variants from the study cohort were merged with reference individuals from the 1000 Genomes Project [5]. For the main Eurasian-focused PCA, European (EUR), South Asian (SAS), and East Asian (EAS) reference populations were included together with the Kazakh, Russian, and Uzbek study groups. A global PCA including African (AFR), Admixed American (AMR), European (EUR), South Asian (SAS), and East Asian (EAS) reference populations was also generated as a supplementary analysis (Supplementary Figure S1).

Before PCA, the study and reference datasets were intersected by shared autosomal variants. Ambiguous, strand-inconsistent, duplicate, and palindromic variants were removed where required for reference-panel harmonization. Missingness filtering, minor allele-frequency filtering, and linkage-disequilibrium pruning were then applied. PCA was performed using PLINK v1.9 [14] and visualized in Python 3 (Python Software Foundation, Beaverton, OR, USA) with Matplotlib 3.11 (NumFOCUS, Austin, TX, USA) [15].

PCA was used only to visualize population structure and provide ancestry context for interpreting pharmacogenomic allele-frequency patterns. It was not used to redefine self-reported population identity, exclude individuals, or infer precise individual ancestry proportions. Because the study was designed as a population-level pharmacogenomic frequency resource rather than a genotype–phenotype association study, principal components were not included as covariates in the allele-frequency calculations.

### 2.4. Selection and Annotation of Clinically Relevant Pharmacogenomic Variants

Clinically relevant pharmacogenomic variants were selected from the PharmGKB ClinPGx clinicalVariants dataset, downloaded in April 2026 [1,2]. ClinPGx is a clinical annotation framework that organizes variant–drug associations and assigns levels of evidence based on the strength of pharmacogenomic support. For the present study, variants annotated with ClinPGx evidence levels 1A, 1B, and 2A were retained because these levels represent established or moderate clinical evidence.

The selected ClinPGx records were filtered by rsID and intersected with the available genotype dataset. Only directly genotyped variants that could be matched by rsID were eligible for inclusion in the frequency analysis. Variants that were absent from the genotype dataset, lacked a matching rsID, or required imputation or complex haplotype reconstruction were excluded. The number of variants retained after this filtering procedure is reported in the Results section.

Pharmacogenomic annotation was based on ClinPGx information, including gene, evidence level, associated drugs, and reported phenotypes. Variants selected for detailed interpretation in the main text were chosen based on clinical evidence level, relevance to major pharmacogenomic drug categories, observed allele frequency, population-frequency differences, and interpretability using directly genotyped variant data. The complete list of all 112 variants is provided in Supplementary Table S1.

### 2.5. Allele and Genotype Frequency Analysis

Allele and genotype frequencies were calculated separately for Kazakh, Russian, and Uzbek groups using PLINK v1.9 [14]. Allele counts were obtained using PLINK --freq, and genotype counts were obtained using PLINK --freqx. For each variant, frequencies were reported for the same selected allele across all three population groups to ensure direct cross-population comparability. This allele is referred to as the reported allele. The reported allele was used only to ensure consistent frequency reporting across population groups. It should not automatically be interpreted as the risk allele, effect allele, decreased-function allele, minor allele, alternative allele, or reference allele unless this direction is supported by the corresponding pharmacogenomic annotation. Therefore, the reported frequencies should be interpreted as frequencies of a fixed allele across groups rather than as population-specific minor allele frequencies. Complete allele and genotype frequency data for all 112 variants, including Hardy–Weinberg equilibrium *p*-values for each population group, are provided in Supplementary Table S1.

To provide an additional genotype-level summary, genotype counts and the proportions of individuals carrying at least one copy of the reported allele were calculated for all selected variants. These summaries were generated for descriptive population-level comparison only and should not be interpreted as complete pharmacogenomic phenotype assignments.

### 2.6. gnomAD v4.1 Reference Frequency Retrieval and Allele Harmonization

To place the study-group allele frequencies within a contemporary global reference framework, all ClinPGx-selected variants retained in the study were evaluated against gnomAD v4.1 (Broad Institute of MIT and Harvard, Cambridge, MA, USA) [12,13]. Ensembl Variant Effect Predictor (VEP; EMBL-European Bioinformatics Institute, Hinxton, UK) [16] was used as an annotation interface to match the study variants and retrieve available gnomAD allele-frequency information; gnomAD was the source of the reference frequency data. Genome and exome allele-frequency data were retained separately because the corresponding datasets differ in sample composition, sequencing strategy, and variant coverage. Frequencies were extracted for the African/African American (AFR), Admixed American (AMR), East Asian (EAS), non-Finnish European (NFE), South Asian (SAS), and Middle Eastern (MID) genetic ancestry groups, when available.

For each variant, the gnomAD allele representation was harmonized with the predefined reported allele used in the study cohort. Alleles were matched by rsID and genomic representation, and strand orientation was checked where necessary. Reverse-complement alleles and deletion alleles represented differently between the study dataset and the reference database were manually verified before frequency comparison. Variants for which no matching gnomAD genome or exome frequency was available for the predefined reported allele were retained in the study-cohort analysis and recorded as having no matching reference frequency for that allele.

The complete comparison of study-group and gnomAD v4.1 frequencies is provided in Supplementary Table S2.

### 2.7. Statistical Analysis

Reported allele frequencies were calculated separately for the Kazakh, Russian, and Uzbek groups. For each variant, 95% confidence intervals were estimated from allele counts using the Clopper–Pearson exact binomial method [17]. Differences in reported allele frequencies between population groups were assessed using two-sided Fisher’s exact tests [18]. Pairwise comparisons were performed for Kazakh versus Russian, Kazakh versus Uzbek, and Russian versus Uzbek groups.

To account for multiple testing, *p*-values from all three pairwise comparisons across the 112 analyzed variants were adjusted using the Benjamini–Hochberg false discovery rate procedure [19]. Statistical significance was defined as an FDR-adjusted q-value < 0.05. Absolute allele-frequency differences and 95% confidence intervals were considered together with the adjusted *p*-values when interpreting differences between the study groups. Complete confidence intervals and pairwise statistical comparison results are provided in Supplementary Table S3.

Hardy–Weinberg equilibrium was assessed separately within each population group using the exact test implemented in PLINK v1.9 [14,20]. These statistical analyses were used for descriptive population-level comparisons and were not intended to generate individual clinical prescribing recommendations.

## 3. Results

### 3.1. Study Cohort and Population Structure

After quality control, 1301 individuals were retained for analysis: 1111 self-reported Kazakh, 156 Russian, and 34 Uzbek individuals. No pair of retained individuals had a PI_HAT value of 0.25 or higher. The overall genotyping rate in the final study dataset was 0.996987, while the autosomal quality-controlled dataset used for population-structure analysis had a total genotyping rate of 0.998275.

**Figure 1:**
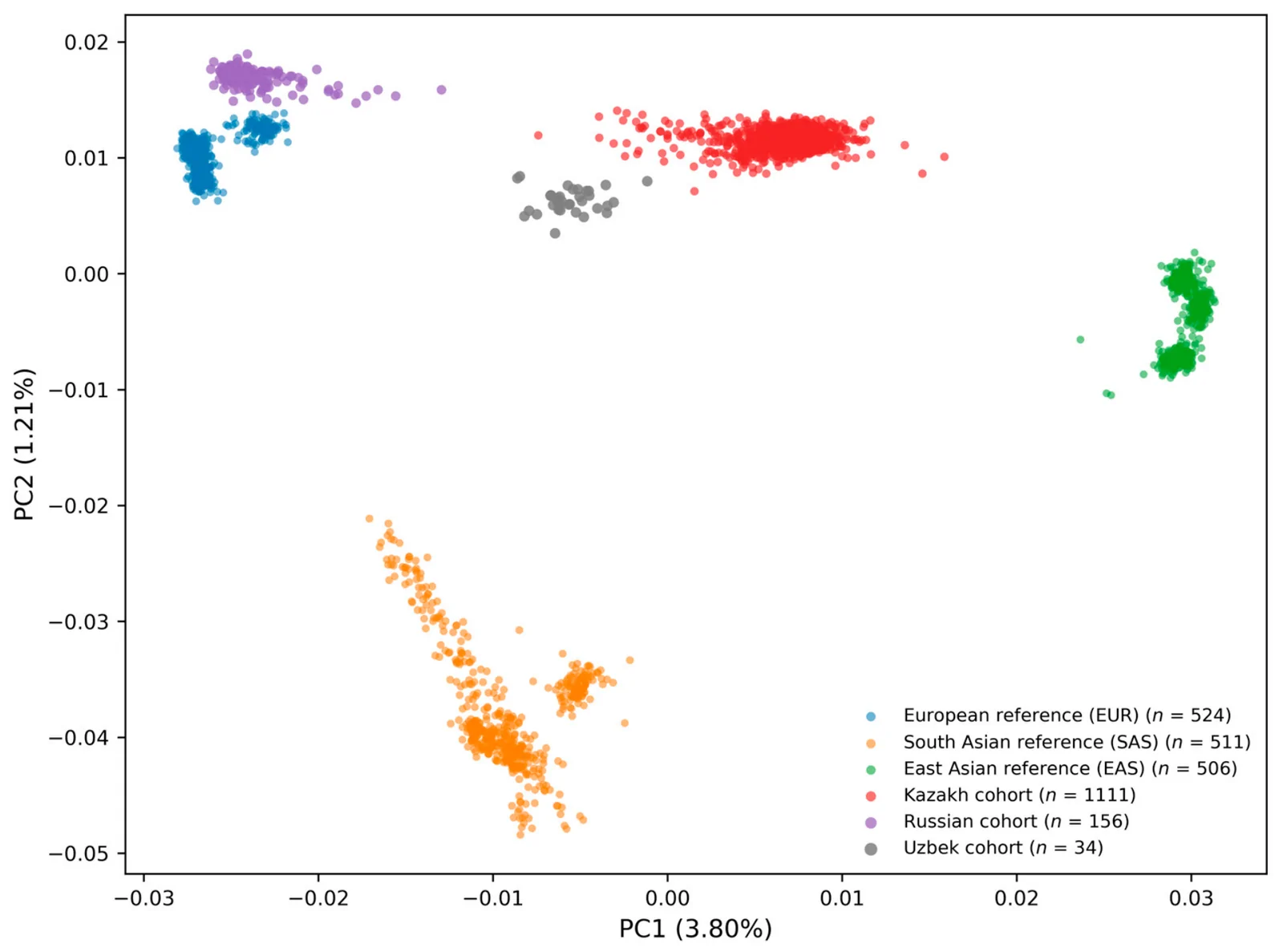
Eurasian-focused reference-based principal component analysis of the study cohort. The analysis included Kazakh, Russian, and Uzbek individuals from the present study together with European (EUR), South Asian (SAS), and East Asian (EAS) reference populations from the 1000 Genomes Project.

The Eurasian-focused PCA included 2842 study and reference individuals and 123,561 LD-pruned autosomal variants. Russian individuals clustered near the European reference population, whereas Kazakh individuals occupied an intermediate position relative to the European, South Asian, and East Asian reference populations. Uzbek individuals were also distributed within the intermediate Eurasian range, although interpretation is limited by the small Uzbek sample size. These patterns provide ancestry context for the subsequent pharmacogenomic allele-frequency comparisons (Figure 1).

### 3.2. ClinPGx-Selected Pharmacogenomic Variant Dataset

A total of 112 directly genotyped ClinPGx-selected variants with evidence levels 1A, 1B, and 2A were retained for analysis. Allele frequencies, genotype counts, and Hardy–Weinberg equilibrium *p*-values for the Kazakh, Russian, and Uzbek groups are provided in Supplementary Table S1. The genotype counts and proportions of individuals carrying at least one copy of the reported allele are provided in Supplementary Table S4.

### 3.3. Hardy–Weinberg Equilibrium Assessment

Hardy–Weinberg equilibrium was assessed separately within each population group for the 112 pharmacogenomic variants. No variant showed nominal deviation from Hardy–Weinberg equilibrium at *p* < 0.05 in the Kazakh group. Two variants, rs2297595 and rs4646437, showed nominal deviation in the Russian group, while *CYP2B6* rs3745274 showed nominal deviation in the Uzbek group. Because these findings were based on unadjusted nominal *p*-values and occurred in the smaller Russian and Uzbek groups, the variants were retained in the descriptive frequency analysis and interpreted cautiously.

### 3.4. Representative Clinically Relevant Pharmacogenomic Variants

Eight representative variants were selected for detailed presentation in the main text (Table 1). Selection was based on ClinPGx evidence level, relevance to major pharmacogenomic drug categories, observed allele frequency, and the magnitude and statistical support of differences between the study groups. Results for all 112 variants, including allele frequencies, genotype counts, confidence intervals, and pairwise statistical comparisons, are provided in Supplementary Tables S1 and S3.

*NUDT15* rs116855232 showed one of the clearest population-frequency differences among the selected variants. The reported T allele frequency was higher in Kazakh individuals than in Russian and Uzbek individuals, and the Kazakh–Russian difference remained significant after false discovery rate correction. This variant is relevant to thiopurine pharmacogenomics and therefore represents an important example of a clinically relevant allele with population-specific frequency variation in Kazakhstan.

Several variants related to drug metabolism, transport, and anticoagulant response also showed notable patterns. *SLCO1B1* rs4149056, *VKORC1* rs9934438, and *UGT1A1* rs10929302 showed FDR-significant Kazakh–Russian differences. *VKORC1* rs9934438 showed the largest absolute frequency difference among the selected variants, with a substantially higher reported G allele frequency in Russians than in Kazakhs. In contrast, *CYP4F2* rs2108622, *ABCG2* rs2231142, and *CYP2B6* rs3745274 were observed at broadly comparable frequencies across the three study groups and did not show FDR-significant pairwise differences.

*DPYD* rs67376798 was rare in all three study groups. Because of the low allele counts and the small Uzbek sample size, frequency estimates for rare variants should be interpreted cautiously and together with their confidence intervals. This is particularly important for variants with observed frequencies close to zero, where the absence of an observed allele in a small group does not imply true absence in the population.

### 3.5. Comparison with gnomAD v4.1 Reference Frequencies

All 112 ClinPGx-selected variants were queried against gnomAD v4.1 genome and exome datasets. Genome allele-frequency data were available for 76 variants, exome data were available for 95 variants, and at least one matching gnomAD frequency for the predefined reported allele was available for 103 variants. For nine variants, the VEP-based query did not return a matching gnomAD v4.1 genome or exome frequency for the predefined reported allele: *DPYD* rs72549306, *EGFR* rs121434568, *CFTR* rs397508513, rs267606723, and rs121909041, and *RYR1* rs193922753, rs193922807, rs193922876, and rs193922878. At several multiallelic loci, gnomAD contained frequency information for other alternate alleles at the same rsID; therefore, these variants were recorded as lacking a matching gnomAD reference frequency for the specific reported allele rather than as being absent from gnomAD. The corresponding reported alleles were not observed in any of the three study groups. The complete comparison, with genome and exome frequencies reported separately, is provided in Supplementary Table S2.

**Figure 2:**
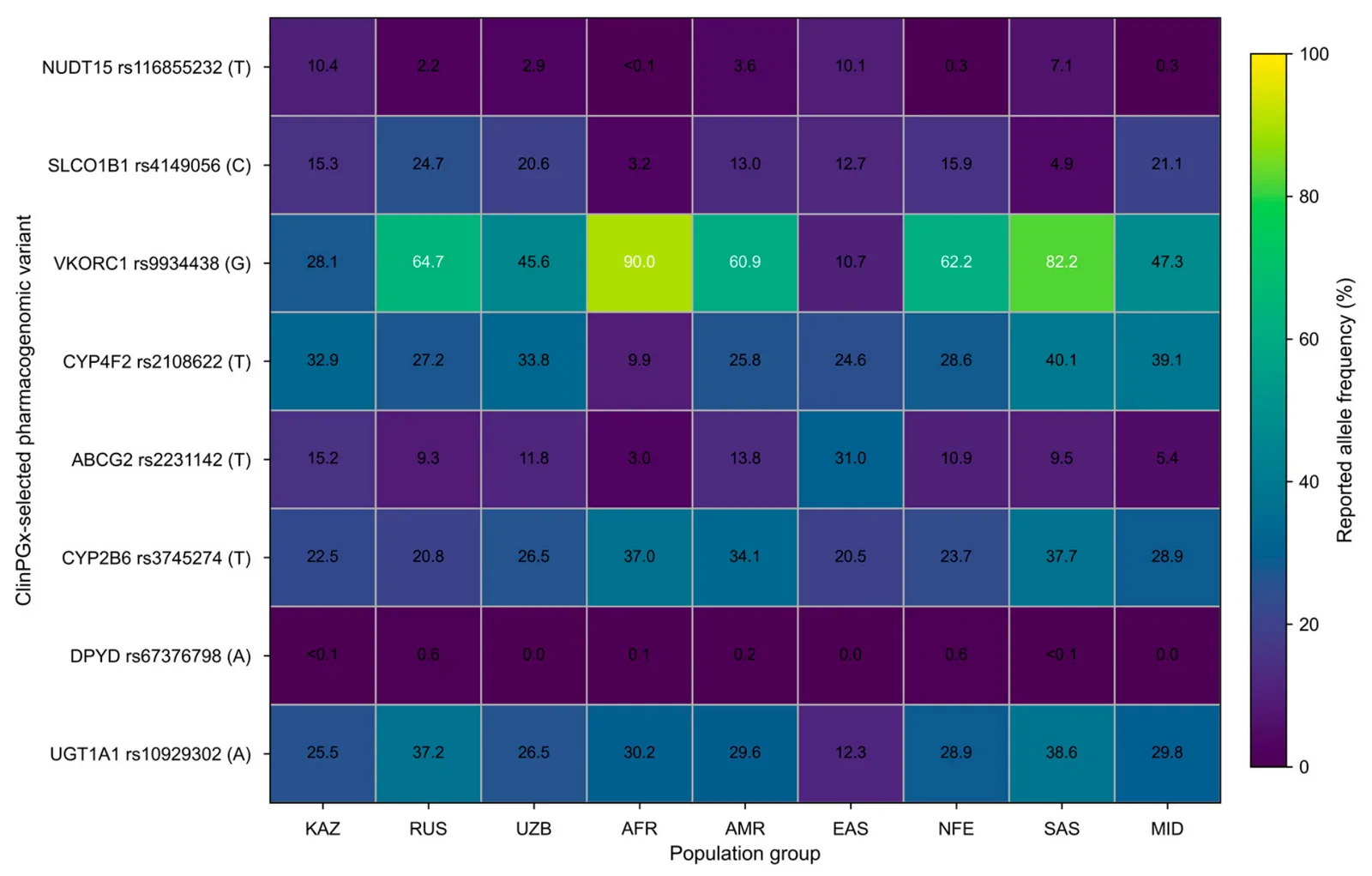
Heatmap of reported allele frequencies for eight selected ClinPGx variants in the Kazakh (KAZ), Russian (RUS), and Uzbek (UZB) study groups and the gnomAD v4.1 genome genetic ancestry groups: African/African American (AFR), Admixed American (AMR), East Asian (EAS), non-Finnish European (NFE), South Asian (SAS), and Middle Eastern (MID). Values represent frequencies (%) of the same predefined reported allele across groups.

The comparison showed substantial variant-specific differences across the study groups and the broad gnomAD genetic ancestry groups. No single gnomAD group consistently approximated the Kazakh, Russian, or Uzbek frequency profiles across all analyzed variants. Figure 2 presents eight representative variants using gnomAD genome frequencies for African/African American (AFR), Admixed American (AMR), East Asian (EAS), non-Finnish European (NFE), South Asian (SAS), and Middle Eastern (MID) groups.

*NUDT15* rs116855232 showed a particularly distinct pattern. The reported T allele frequency in the Kazakh group was 10.36%, which was close to the gnomAD EAS frequency of 10.1% and higher than the frequencies observed in AFR (<0.1%), AMR (3.6%), NFE (0.3%), SAS (7.1%), and MID (0.3%). The corresponding frequencies in the Russian and Uzbek groups were 2.24% and 2.94%, respectively. In contrast, the *VKORC1* rs9934438 reported G allele was more frequent in the Russian group (64.74%) and showed similar frequencies in the gnomAD AMR (60.9%) and NFE (62.2%) groups, whereas its frequency in the Kazakh group was 28.08%. The Uzbek frequency was 45.59%, which was close to the gnomAD MID frequency of 47.3%.

Other variants displayed different cross-population patterns. The SLCO1B1 rs4149056 reported C allele occurred at frequencies of 15.35%, 24.68%, and 20.59% in the Kazakh, Russian, and Uzbek groups, respectively; the Kazakh frequency was close to the gnomAD NFE frequency of 15.9%. The *UGT1A1* rs10929302 reported A allele was more frequent in Russians (37.18%) than in Kazakhs (25.45%) and Uzbeks (26.47%), and the Russian frequency was close to the gnomAD SAS frequency of 38.6%. *CYP4F2* rs2108622 and *CYP2B6* rs3745274 were common across multiple study and reference groups, whereas *DPYD* rs67376798 remained rare in all displayed groups.

These findings demonstrate that pharmacogenomic frequency patterns in Kazakhstan are locus-specific and should not be extrapolated from a single broad reference group. The comparisons describe reported-allele frequencies and should not be interpreted as direct measures of ancestry or as individual pharmacogenomic phenotype assignments.

## 4. Discussion

### 4.1. Population-Specific Pharmacogenomic Variation in Kazakhstan

This study provides a ClinPGx-prioritized pharmacogenomic frequency resource for Kazakh, Russian, and Uzbek population groups residing in Kazakhstan. By analyzing the three groups within the same framework and placing the observed frequencies in the context of gnomAD v4.1, the study provides a consistent population-level characterization of pharmacogenomic variation in Kazakhstan.

The observed pharmacogenomic frequency patterns varied among the three study groups, with some variants showing statistically supported population differences and others showing broadly comparable frequencies. These findings support population-specific reporting rather than a single combined national estimate, while estimates for the smaller Uzbek group should be interpreted cautiously.

Beyond the observed allele-frequency differences, the combined presentation of allele frequencies, genotype counts, confidence intervals, and the proportions of individuals carrying at least one copy of the reported allele provides a broader descriptive view of pharmacogenomic variation in Kazakhstan. Together with the gnomAD comparison, these results may help identify gene–drug areas that warrant local validation and implementation studies, particularly where international pharmacogenomic guidelines are available but population-specific data from Kazakhstan and Central Asia remain limited.

### 4.2. Clinical Relevance of Selected Pharmacogenomic Variants

*NUDT15* rs116855232 was one of the most notable findings because it showed a clear population-specific pattern in the Kazakh group. The higher frequency observed in Kazakhs compared with Russians and Uzbeks suggests that *NUDT15*-related thiopurine pharmacogenomics may be particularly relevant for future population-informed studies in Kazakhstan. This interpretation is supported by the established role of *NUDT15* in thiopurine response, with CPIC providing genotype-guided recommendations for thiopurine therapy based on *TPMT* and *NUDT15* genotypes [21]. Evidence from thiopurine-treated patients further supports the clinical relevance of *NUDT15* c.415C>T, which has been associated with increased risk of thiopurine-induced myelosuppression and treatment intolerance [22]. Therefore, the observed frequency pattern highlights the importance of considering *NUDT15* variation when developing pharmacogenomic resources for Kazakhstan.

Anticoagulant-related variants also showed important population-specific patterns. *VKORC1* rs9934438 showed one of the strongest differences among the selected variants, with a substantially higher reported G allele frequency in Russians than in Kazakhs. This finding is relevant because *VKORC1* is a major pharmacodynamic determinant of warfarin response, and *VKORC1* haplotypes have been shown to stratify patients into different warfarin dose-requirement groups [23]. In addition, the International Warfarin Pharmacogenetics Consortium demonstrated that algorithms incorporating clinical and genetic factors improve warfarin dose estimation compared with clinical variables alone [24]. *CYP4F2* rs2108622 was common in all three study groups and is also relevant to warfarin pharmacogenomics, as this variant has been associated with altered warfarin dose requirements [25]. CPIC guidelines include *CYP2C9*, *VKORC1*, and *CYP4F2* in pharmacogenetics-guided warfarin dosing [26]. The recent Kazakh genomic landscape study also highlighted *CYP4F2* rs2108622 as a biomedically relevant pharmacogenomic variant showing a distinctive frequency pattern in Kazakh individuals [11]. Our findings provide additional population-specific context by reporting this variant across Kazakh, Russian, and Uzbek groups residing in Kazakhstan.

Transporter-related pharmacogenomic variants were also observed at clinically relevant frequencies in the study groups. *SLCO1B1* rs4149056 is a well-established pharmacogene for statin-associated musculoskeletal symptoms, and current CPIC guidelines include *SLCO1B1*, *ABCG2*, and *CYP2C9* in recommendations related to statin-associated adverse effects [27]. The *ABCG2* variant rs2231142 was also detected at appreciable frequencies across the study groups. Together, these findings indicate that transporter-related pharmacogenomic variation is relevant for population-level pharmacogenomic profiling in Kazakhstan. These data may be useful for future studies of cardiovascular and metabolic pharmacotherapy, but clinical interpretation should remain gene–drug specific and should not be generalized across all statins or transporter substrates.

Other clinically relevant variants showed different patterns across the study groups. *CYP2B6* rs3745274 was common and broadly comparable across Kazakh, Russian, and Uzbek individuals, indicating that not all pharmacogenomic variants display strong population differentiation within Kazakhstan. This variant is clinically relevant for efavirenz-containing antiretroviral therapy, and CPIC provides recommendations for efavirenz prescribing based on *CYP2B6* genotype [28]. *DPYD* rs67376798 was rare in all three study groups. Although rare, *DPYD* variation is clinically important because reduced dihydropyrimidine dehydrogenase activity can increase the risk of severe fluoropyrimidine toxicity, and CPIC provides recommendations for fluoropyrimidine dosing based on *DPYD* genotype [29]. The wide confidence intervals for low-frequency variants, especially in the Uzbek group, show why rare-variant estimates should be interpreted cautiously. *UGT1A1*-related rs10929302 was common in all three groups; however, clinical interpretation of *UGT1A1* often depends on haplotypes and repeat polymorphisms that may not be fully captured by microarray data. Therefore, the *UGT1A1* findings should be viewed as part of a broader allele-frequency resource rather than a complete assessment of *UGT1A1* functional variation [30]. Among the nine variants for which no matching gnomAD frequency was returned for the predefined reported allele, several have clinically relevant allele-specific consequences despite their absence from the present study groups. *DPYD* rs72549306 (DPYD*11; p.Val335Leu) was originally reported in a patient with decreased DPD activity and severe 5-fluorouracil toxicity, although a later functional study did not demonstrate a significant reduction in enzyme activity, indicating that the functional evidence for this rare variant is not fully consistent [31,32]. *EGFR* rs121434568 (p.Leu858Arg; L858R) is a well-characterized activating mutation in non-small-cell lung cancer associated with sensitivity to *EGFR* tyrosine kinase inhibitors such as gefitinib [33]. Because the present study analyzed population-level genotype data rather than tumor sequencing data, the absence of this reported allele in our cohort should not be interpreted as evidence regarding its occurrence in tumors. Among the *CFTR* variants, rs397508513 (p.Lys1060Thr) has shown increased chloride transport in response to ivacaftor in functional studies [34], rs267606723 with the reported T allele corresponds to p.Gly1244Val, a rare missense variant originally described in a patient with cystic fibrosis [35], and rs121909041 (p.Ser1255Pro) has demonstrated ivacaftor-responsive channel activity in vitro [36]. The *RYR1* variants rs193922753 (p.Arg163Leu), rs193922807 (p.Gly2375Ala), rs193922876 (p.His4833Tyr), and rs193922878 (p.Leu4838Val) have been reported in association with malignant-hyperthermia susceptibility, with clinical and/or functional evidence supporting altered *RYR1* calcium-channel responses [37,38,39,40]. None of these reported alleles were observed in the present study groups; however, their absence from this cohort should not be interpreted as evidence that they are absent from the populations of Kazakhstan.

### 4.3. Comparison with Previous Studies and Reference Populations

Our study extends previous pharmacogenetic and genomic research in Kazakhstan by applying a clinically prioritized ClinPGx-based selection strategy to three population groups residing in the country. Earlier studies provided important information on selected ADME-related variants, pharmacogenetic predictors of treatment response, and the broader genomic landscape of Kazakh individuals [9,10,11]. The present study complements this work by reporting allele and genotype frequencies for 112 directly genotyped ClinPGx-selected variants in Kazakh, Russian, and Uzbek groups and by evaluating these variants against the contemporary gnomAD v4.1 genome and exome reference datasets [12,13].

Overall, the gnomAD comparison reinforced the locus-specific nature of the observed pharmacogenomic frequency patterns and showed that no single broad reference group consistently represented all three study populations.

The gnomAD comparisons should nevertheless be interpreted cautiously. The gnomAD categories represent broad genetic ancestry groups and are not equivalent to nationality, self-reported ethnicity, or geographically defined Central Asian populations [13]. Differences in sample composition, sequencing strategy, variant coverage, and allele representation may also affect comparisons between the study cohort and gnomAD. For this reason, genome and exome frequencies were analyzed separately, and the reference data were used to provide population-level context rather than to assign ancestry or predict individual pharmacogenomic phenotypes. These considerations further support the collection of directly measured pharmacogenomic data from Kazakhstan and other underrepresented Central Asian populations.

### 4.4. Strengths of the Study

Several strengths of this study should be noted. First, the relatively large Kazakh cohort enabled detailed estimation of allele and genotype frequencies for a clinically prioritized set of pharmacogenomic variants. Second, variant selection was based on predefined ClinPGx evidence levels, providing a transparent and reproducible prioritization framework. Third, frequencies were reported for the same predefined allele across all study and reference groups, thereby avoiding comparisons based on population-specific minor alleles that might differ between populations. Fourth, the inclusion of Russian and Uzbek individuals allowed pharmacogenomic variation within Kazakhstan to be characterized beyond the largest population group. Fifth, all 112 selected variants were systematically queried against contemporary gnomAD v4.1 genome and exome datasets, with the two datasets retained separately to avoid inappropriate pooling. Finally, the study included 95% confidence intervals, FDR-adjusted pairwise comparisons, Hardy–Weinberg equilibrium assessment, genotype counts, and the proportions of individuals carrying at least one copy of the reported allele, providing multiple complementary measures for interpreting the observed population-level frequency patterns.

### 4.5. Limitations

This study has several limitations. First, the study groups were imbalanced in sample size. The Kazakh group was substantially larger than the Russian and Uzbek groups, and the Uzbek group was small (*n* = 34). Therefore, frequency estimates for Uzbek individuals, especially for rare variants, should be considered exploratory and interpreted together with their confidence intervals. For variants with very low observed allele counts, absence of an allele in a small group should not be interpreted as true absence in the population.

Second, population group assignment was based on self-reported population identity recorded in the available sample metadata. Reference-based PCA was added to provide ancestry context for the interpretation of pharmacogenomic frequency patterns, but PCA was not used to redefine population identity, infer precise individual ancestry proportions, or exclude individuals. Because this study was designed as a population-level frequency resource rather than a genotype–phenotype association study, principal components were not included as covariates in the allele-frequency comparisons.

Third, the analysis was based on array-based genotype data and therefore does not capture the full spectrum of pharmacogenomic variation. Important variant types not fully assessed include star alleles, structural variants, copy-number variants, HLA alleles, repeat polymorphisms, and complex haplotypes. This limitation is especially relevant for genes such as *CYP2D6*, *CYP2C19*, *UGT1A1*, and other pharmacogenes where clinical interpretation often requires haplotype or copy-number calling.

Fourth, the predefined reported allele was selected to ensure consistent frequency reporting across the study and reference groups. It should not automatically be interpreted as the risk, effect, decreased-function, reference, alternative, or minor allele unless this interpretation is supported by the corresponding pharmacogenomic annotation. Although all 112 selected variants were queried against gnomAD v4.1, at least one matching genome or exome frequency for the predefined reported allele was available for 103 variants, whereas no matching reference frequency for that allele was available for nine variants. In addition, the gnomAD categories represent broad genetic ancestry groups and should not be treated as direct proxies for self-reported ethnicity, nationality, or Central Asian populations. Differences in sample composition, sequencing strategy, variant coverage, and allele representation may also influence comparisons between the study cohort and gnomAD. Genome and exome frequencies were therefore retained as separate reference datasets rather than combined into a pooled estimate.

Finally, this study reports population-level allele and genotype frequencies, together with the proportions of individuals carrying at least one copy of the predefined reported allele, but it does not evaluate drug response, adverse drug reactions, therapeutic dose requirements, or other clinical outcomes. The results should therefore be interpreted as a descriptive pharmacogenomic frequency resource rather than as individual prescribing recommendations or complete pharmacogenomic phenotype assignments. Future studies integrating comprehensive pharmacogene profiling with treatment and outcome data will be required before these findings can directly support clinical pharmacogenomic implementation in Kazakhstan.

### 4.6. Future Directions

Future pharmacogenomic studies in Kazakhstan and Central Asia should advance in several complementary directions. First, larger and more balanced cohorts are needed, particularly for Uzbek and other underrepresented population groups, to improve the precision of frequency estimates for rare and clinically important variants. Second, sequencing-based or targeted pharmacogene profiling should be used to resolve star alleles, copy-number variation, structural variants, HLA alleles, repeat polymorphisms, and complex haplotypes that cannot be comprehensively assessed using array-based genotype data. Third, population-level frequency resources should be integrated with clinical information, including drug response, adverse drug reactions, therapeutic dose requirements, and treatment outcomes. Finally, future studies should include additional geographically and genetically relevant Central Asian reference populations, because broad gnomAD genetic ancestry groups cannot substitute for directly sampled regional populations. Together, these approaches would support the transition from descriptive frequency comparisons to validated genotype–phenotype interpretation and evidence-based pharmacogenomic implementation in Kazakhstan.

## 5. Conclusions

This study provides a clinically prioritized pharmacogenomic frequency resource for Kazakh, Russian, and Uzbek population groups residing in Kazakhstan. A total of 112 directly genotyped ClinPGx-selected variants with evidence levels 1A, 1B, and 2A were analyzed for population-specific allele and genotype frequencies. Several clinically relevant variants showed marked differences between the study groups, including *NUDT*15 rs116855232, *SLCO1B1* rs4149056, *VKORC1* rs9934438, and *UGT1A1* rs10929302, whereas *CYP4F2* rs2108622 and *CYP2B6* rs3745274 were observed at broadly comparable frequencies across the three groups.

All 112 selected variants were queried against the gnomAD v4.1 genome and exome datasets, and at least one matching reference frequency for the predefined reported allele was available for 103 variants. The comparison showed that the observed pharmacogenomic frequency patterns were locus-specific and could not be consistently represented by a single broad gnomAD genetic ancestry group. These findings support the direct characterization and separate reporting of pharmacogenomic variation in populations from Kazakhstan rather than extrapolation from broad continental reference categories.

The results should be interpreted as a descriptive population-level resource rather than as individual prescribing recommendations or complete pharmacogenomic phenotype assignments. The study did not assess drug response, adverse drug reactions, treatment outcomes, or complete pharmacogene haplotypes, and the small Uzbek sample size limits the precision of frequency estimates, particularly for rare variants. Larger and more balanced cohorts, comprehensive pharmacogene profiling, regional Central Asian reference datasets, and clinical outcome data will be required to support future pharmacogenomic implementation in Kazakhstan and Central Asia.

## Figures and Tables

**Table 1:** Representative clinically relevant pharmacogenomic variants selected for detailed interpretation.

Gene	SNP	Reported Allele	KAZ % (95% CI)	RUS % (95% CI)	UZB % (95% CI)	FDR-Significant Pairwise Comparison
*NUDT15*	rs116855232	T	10.36 (9.12–11.70)	2.24 (0.91–4.57)	2.94 (0.36–10.22)	KAZ–RUS
*SLCO1B1*	rs4149056	C	15.35 (13.87–16.91)	24.68 (20–29.85)	20.59 (11.74–32.12)	KAZ–RUS
*VKORC1*	rs9934438	G	28.08 (26.22–30.00)	64.74 (59.16–70.00)	45.59 (33.45–58.12)	KAZ–RUS
*CYP4F2*	rs2108622	T	32.90 (30.95–34.90)	27.24 (22.38–32.55)	33.82 (22.79–46.32)	NS
*ABCG2*	rs2231142	T	15.21 (13.74–16.77)	9.29 (6.31–13.08)	11.76 (5.22–21.87)	NS
*CYP2B6*	rs3745274	T	22.45 (20.73–24.25)	20.78 (16.39–25.74)	26.47 (16.50–38.57)	NS
*DPYD*	rs67376798	A	0.05 (0.00–0.25)	0.64 (0.08–2.30)	0.00 (0.00–5.28)	NS
*UGT1A1*	rs10929302	A	25.45 (23.65–27.32)	37.18 (31.80–42.80)	26.47 (16.50–38.57)	KAZ–RUS

Note: Values are reported-allele frequencies expressed as percentages with 95% confidence intervals. The same predefined reported allele was used for all three population groups. FDR significance was defined as a Benjamini–Hochberg-adjusted q-value < 0.05. NS, not significant after FDR correction.

## Data Availability

The summary data generated and analyzed during the present study are provided in the Supplementary Materials. Individual-level genotype data are not publicly available due to ethical and privacy restrictions related to human genetic data. Additional information may be made available from the corresponding author upon reasonable request and subject to institutional and ethical approvals.

## Supplementary Materials

The following supporting information can be downloaded at: https://www.mdpi.com/article/10.3390/biology15171477/s1, Supplementary Tables S1–S4 are provided as separate worksheets in a single Excel workbook (Supplementary_Tables_S1-S4.xlsx). Supplementary Table S1: Allele and genotype frequencies of 112 ClinPGx-selected pharmacogenomic variants in Kazakh, Russian, and Uzbek population groups residing in Kazakhstan, including Hardy–Weinberg equilibrium *p*-values for each group. Supplementary Table S2: Comparison of reported allele frequencies for all 112 ClinPGx-selected variants in the study groups with available gnomAD v4.1 genome and exome frequencies; genome and exome data are presented separately for the corresponding genetic ancestry groups. Supplementary Table S3: Reported allele frequencies with 95% confidence intervals and pairwise statistical comparisons between Kazakh, Russian, and Uzbek population groups. Supplementary Table S4: Genotype counts and proportions of individuals carrying at least one copy of the predefined reported allele for all 112 ClinPGx-selected pharmacogenomic variants. Supplementary Figure S1: Global reference-based principal component analysis including Kazakh, Russian, and Uzbek individuals from the present study together with African, Admixed American, East Asian, European, and South Asian reference populations from the 1000 Genomes Project.

## Abbreviations

The following abbreviations are used in this manuscript:

AFR	African/African American
AMR	Admixed American
CPIC	Clinical Pharmacogenetics Implementation Consortium
DPWG	Dutch Pharmacogenetics Working Group
EAS	East Asian
EUR	European
FDR	False discovery rate
gnomAD	Genome Aggregation Database
GRCh38	Genome Reference Consortium Human Build 38
HWE	Hardy–Weinberg equilibrium
KAZ	Kazakh
LD	Linkage disequilibrium
MID	Middle Eastern
NFE	Non-Finnish European
PCA	Principal component analysis
RUS	Russian
SAS	South Asian
UZB	Uzbek
VEP	Variant Effect Predictor

## References

[1] Whirl-Carrillo M., Huddart R., Gong L., Sangkuhl K., Thorn C.F., Whaley R., Klein T.E. (2021). An Evidence-Based Framework for Evaluating Pharmacogenomics Knowledge for Personalized Medicine. Clin. Pharmacol. Ther..

[2] Gong L., Whirl-Carrillo M., Klein T.E. (2021). PharmGKB, an Integrated Resource of Pharmacogenomic Knowledge. Curr. Protoc..

[3] Li B., Sangkuhl K., Whaley R., Woon M., Keat K., Whirl-Carrillo M., Ritchie M.D., Klein T.E. (2023). Frequencies of Pharmacogenomic Alleles across Biogeographic Groups in a Large-Scale Biobank. Am. J. Hum. Genet..

[4] Huddart R., Fohner A.E., Whirl-Carrillo M., Wojcik G.L., Gignoux C.R., Popejoy A.B., Bustamante C.D., Altman R.B., Klein T.E. (2019). Standardized Biogeographic Grouping System for Annotating Populations in Pharmacogenetic Research. Clin. Pharmacol. Ther..

[5] The 1000 Genomes Project Consortium (2015). A Global Reference for Human Genetic Variation. Nature.

[6] Magavern E.F., Gurdasani D., Ng F.L., Lee S.S.-J. (2022). Health Equality, Race and Pharmacogenomics. Br. J. Clin. Pharmacol..

[7] Tremmel R., Zhou Y., Camara M.D., Laarif S., Eliasson E., Lauschke V.M. (2025). PharmFreq: A Comprehensive Atlas of Ethnogeographic Allelic Variation in Clinically Important Pharmacogenes. Nucleic Acids Res..

[8] Bureau of National Statistics of the Agency for Strategic Planning and Reforms of the Republic of Kazakhstan Thematic Collection on the Ethnic Composition of the Population Based on the Results of the 2021 Census. https://stat.gov.kz/ru/news/byuro-natsionalnoy-statistiki-opublikovalo-tematicheskiy-sbornik-po-natsionalnomu-sostavu-naseleniya/.

[9] Iskakova A.N., Romanova A.A., Aitkulova A.M., Sikhayeva N.S., Zholdybayeva E.V., Ramanculov E.M. (2016). Polymorphisms in Genes Involved in the Absorption, Distribution, Metabolism, and Excretion of Drugs in the Kazakhs of Kazakhstan. BMC Genet..

[10] Imangaliyeva A.T., Sikhayeva N.S., Baidurin S.A., Suleimenova B.A., Romanova A.A., Zholdybayeva E.V., Shaimerdenov S.A., Kuanysheva A.S., Bolatov A. (2025). Pharmacogenetic Predictors of Metformin Response in Metabolic Syndrome and Type 2 Diabetes: Evidence from a Cohort Study in Kazakhstan. J. Diabetes Res..

[11] Serikzhan A., Daniyarov A., Molkenov A., Akhmetova A., Abilova Z., Sharip A., Yerezhepov D., Rakhimova S., Kozhamkulov U., Kushugulova A. (2025). Genomic Landscape of the Great Steppe: Genetic Variants in Healthy Kazakh Individuals. Sci. Data.

[12] Chen S., Francioli L.C., Goodrich J.K., Collins R.L., Kanai M., Wang Q., Alföldi J., Watts N.A., Vittal C., Gauthier L.D. (2024). A Genomic Mutational Constraint Map Using Variation in 76,156 Human Genomes. Nature.

[13] Genome Aggregation Database (gnomAD) gnomAD v4.1. https://gnomad.broadinstitute.org/news/2024-04-gnomad-v4-1/.

[14] Chang C.C., Chow C.C., Tellier L.C., Vattikuti S., Purcell S.M., Lee J.J. (2015). Second-Generation PLINK: Rising to the Challenge of Larger and Richer Datasets. GigaScience.

[15] Hunter J.D. (2007). Matplotlib: A 2D Graphics Environment. Comput. Sci. Eng..

[16] McLaren W., Gil L., Hunt S.E., Riat H.S., Ritchie G.R.S., Thormann A., Flicek P., Cunningham F. (2016). The Ensembl Variant Effect Predictor. Genome Biol..

[17] Clopper C.J., Pearson E.S. (1934). The Use of Confidence or Fiducial Limits Illustrated in the Case of the Binomial. Biometrika.

[18] Fisher R.A. (1922). On the Interpretation of χ^2^ from Contingency Tables, and the Calculation of P. J. R. Stat. Soc..

[19] Benjamini Y., Hochberg Y. (1995). Controlling the False Discovery Rate: A Practical and Powerful Approach to Multiple Testing. J. R. Stat. Soc. Ser. B.

[20] Wigginton J.E., Cutler D.J., Abecasis G.R. (2005). A Note on Exact Tests of Hardy–Weinberg Equilibrium. Am. J. Hum. Genet..

[21] Relling M.V., Schwab M., Whirl-Carrillo M., Suarez-Kurtz G., Pui C.-H., Stein C.M., Moyer A.M., Evans W.E., Klein T.E., Antillon-Klussmann F.G. (2019). Clinical Pharmacogenetics Implementation Consortium (CPIC) Guideline for Thiopurine Dosing Based on TPMT and NUDT15 Genotypes: 2018 Update. Clin. Pharmacol. Ther..

[22] Du S., Huang X., He X., Mao M., Chen M., Zhang R., Shao H., Lv Z., Liu X., Chuan J. (2024). Association of NUDT15 Gene Polymorphism with Adverse Reaction, Treatment Efficacy, and Dose of 6-Mercaptopurine in Patients with Acute Lymphoblastic Leukemia: A Systematic Review and Meta-Analysis. Haematologica.

[23] Rieder M.J., Reiner A.P., Gage B.F., Nickerson D.A., Eby C.S., McLeod H.L., Blough D.K., Thummel K.E., Veenstra D.L., Rettie A.E. (2005). Effect of VKORC1 Haplotypes on Transcriptional Regulation and Warfarin Dose. N. Engl. J. Med..

[24] Klein T.E., Altman R.B., Eriksson N., Gage B.F., Kimmel S.E., Lee M.-T.M., Limdi N.A., Page D., Roden D.M., International Warfarin Pharmacogenetics Consortium (2009). Estimation of the Warfarin Dose with Clinical and Pharmacogenetic Data. N. Engl. J. Med..

[25] Caldwell M.D., Awad T., Johnson J.A., Gage B.F., Falkowski M., Gardina P., Hubbard J., Turpaz Y., Langaee T.Y., Eby C. (2008). CYP4F2 Genetic Variant Alters Required Warfarin Dose. Blood.

[26] Johnson J.A., Caudle K.E., Gong L., Whirl-Carrillo M., Stein C.M., Scott S.A., Lee M.T., Gage B.F., Kimmel S.E., Perera M.A. (2017). Clinical Pharmacogenetics Implementation Consortium (CPIC) Guideline for Pharmacogenetics-Guided Warfarin Dosing: 2017 Update. Clin. Pharmacol. Ther..

[27] Cooper-DeHoff R.M., Niemi M., Ramsey L.B., Luzum J.A., Tarkiainen E.K., Straka R.J., Gong L., Tuteja S., Wilke R.A., Wadelius M. (2022). The Clinical Pharmacogenetics Implementation Consortium Guideline for SLCO1B1, ABCG2, and CYP2C9 Genotypes and Statin-Associated Musculoskeletal Symptoms. Clin. Pharmacol. Ther..

[28] Desta Z., Gammal R.S., Gong L., Whirl-Carrillo M., Gaur A.H., Sukasem C., Hockings H., Myers A., Swart M., Tyndale R.F. (2019). Clinical Pharmacogenetics Implementation Consortium (CPIC) Guideline for CYP2B6 and Efavirenz-Containing Antiretroviral Therapy. Clin. Pharmacol. Ther..

[29] Amstutz U., Henricks L.M., Offer S.M., Barbarino J., Schellens J.H.M., Swen J.J., Klein T.E., McLeod H.L., Caudle K.E., Diasio R.B. (2018). Clinical Pharmacogenetics Implementation Consortium (CPIC) Guideline for Dihydropyrimidine Dehydrogenase Genotype and Fluoropyrimidine Dosing: 2017 Update. Clin. Pharmacol. Ther..

[30] Hulshof E.C., Deenen M.J., Nijenhuis M., Soree B., de Boer-Veger N.J., Buunk A.-M., Houwink E.J.F., Risselada A., Rongen G.A.P.J.M., van Schaik R.H.N. (2023). Dutch Pharmacogenetics Working Group (DPWG) Guideline for the Gene–Drug Interaction between UGT1A1 and Irinotecan. Eur. J. Hum. Genet..

[31] Kouwaki M., Hamajima N., Sumi S., Nonaka M., Sasaki M., Dobashi K., Kidouchi K., Togari H., Wada Y. (1998). Identification of Novel Mutations in the Dihydropyrimidine Dehydrogenase Gene in a Japanese Patient with 5-Fluorouracil Toxicity. Clin. Cancer Res..

[32] Offer S.M., Fossum C.C., Wegner N.J., Stuflesser A.J., Butterfield G.L., Diasio R.B. (2014). Comparative Functional Analysis of DPYD Variants of Potential Clinical Relevance to Dihydropyrimidine Dehydrogenase Activity. Cancer Res..

[33] Lynch T.J., Bell D.W., Sordella R., Gurubhagavatula S., Okimoto R.A., Brannigan B.W., Harris P.L., Haserlat S.M., Supko J.G., Haluska F.G. (2004). Activating Mutations in the Epidermal Growth Factor Receptor Underlying Responsiveness of Non-Small-Cell Lung Cancer to Gefitinib. N. Engl. J. Med..

[34] Van Goor F., Yu H., Burton B., Hoffman B.J. (2014). Effect of Ivacaftor on CFTR Forms with Missense Mutations Associated with Defects in Protein Processing or Function. J. Cyst. Fibros..

[35] Savov A., Jordanova A., Gavrilov D., Angelicheva D., Kalaydjieva L. (1994). G1244V: A Novel Missense Mutation in Exon 20 of the CFTR Gene in a Bulgarian Cystic Fibrosis Patient. Hum. Mol. Genet..

[36] Yu H., Burton B., Huang C.-J., Worley J., Cao D., Johnson J.P., Urrutia A., Joubran J., Seepersaud S., Sussky K. (2012). Ivacaftor Potentiation of Multiple CFTR Channels with Gating Mutations. J. Cyst. Fibros..

[37] Monnier N., Kozak-Ribbens G., Krivosic-Horber R., Nivoche Y., Qi D., Kraev N., Loke J., Sharma P., Tegazzin V., Figarella-Branger D. (2005). Correlations between Genotype and Pharmacological, Histological, Functional, and Clinical Phenotypes in Malignant Hyperthermia Susceptibility. Hum. Mutat..

[38] Wehner M., Rueffert H., Koenig F., Olthoff D. (2003). Calcium Release from Sarcoplasmic Reticulum Is Facilitated in Human Myotubes Derived from Carriers of the Ryanodine Receptor Type 1 Mutations Ile2182Phe and Gly2375Ala. Genet. Test..

[39] Anderson A.A., Brown R.L., Polster B., Pollock N., Stowell K.M. (2008). Identification and Biochemical Characterization of a Novel Ryanodine Receptor Gene Mutation Associated with Malignant Hyperthermia. Anesthesiology.

[40] Oyamada H., Oguchi K., Saitoh N., Yamazawa T., Hirose K., Kawana Y., Wakatsuki K., Oguchi K., Tagami M., Hanaoka K. (2002). Novel Mutations in C-Terminal Channel Region of the Ryanodine Receptor in Malignant Hyperthermia Patients. Jpn. J. Pharmacol..

